# Prediction of Fatigue Crack Growth Rate Based on Entropy Generation

**DOI:** 10.3390/e22010009

**Published:** 2019-12-19

**Authors:** Roslinda Idris, Shahrum Abdullah, Prakash Thamburaja, Mohd Zaidi Omar

**Affiliations:** Department of Mechanical and Manufacturing Engineering, Faculty of Engineering and Built Environment, Universiti Kebangsaan Malaysia, UKM Bangi 43600, Selangor, Malaysia

**Keywords:** degradation-entropy generation theorem, dual-phase steel, entropy generation, fatigue crack growth rate, spectrum loading

## Abstract

This paper presents the assessment of fatigue crack growth rate for dual-phase steel under spectrum loading based on entropy generation. According to the second law of thermodynamics, fatigue crack growth is related to entropy gain because of its irreversibility. In this work, the temperature evolution and crack length were simultaneously measured during fatigue crack growth tests until failure to ensure the validity of the assessment. Results indicated a significant correlation between fatigue crack growth rate and entropy. This relationship is the basis in developing a model that can determine the characteristics of fatigue crack growth rates, particularly under spectrum loading. Predictive results showed that the proposed model can accurately predict the fatigue crack growth rate under spectrum loading in all cases. The root mean square error in all cases is 10^−7^ m/cycle. In conclusion, entropy generation can accurately predict the fatigue crack growth rate of dual-phase steels under spectrum loading.

## 1. Introduction

Fatigue involves crack initiation, propagation and final fracture. The fatigue cracking problem of mechanical structures and components are exposed to variable amplitude loading (VAL) [1,2,3,4]. The number of cycles required for crack growth to reach a certain distance or failure can be predicted based on the principle of fracture mechanics. Paris and Erdogan [5] first formulated the empirical correlations between the fatigue crack growth (FCG) rate *da*/*dN* and the stress intensity factor range Δ*K* using a simple power law. Since then, fracture mechanics has provided exceptional contributions to the improvement of FCG prediction. The mathematical modelling of the FCG rate for steel is necessary to predict the residual strength or remaining life. The FCG in steel is a complex process with irreversible changes at micro, meso and macrolevels. Other classical approaches in determining the FCG rate are highly empirical in nature [6,7]. On the one hand, an empirical model simply describes a data but does not derive from physical principles. On the other hand, a model that fits a set of data cannot explain the reason for the material response. Hence, a model based on fundamental physical principles, including thermodynamics, is more necessary than a model based on analogy [8]. Any material behaviour can be expressed as a mathematical model when the second law of thermodynamics is fulfilled with suitable selection state variables, analytical expressions of the state potential and dissipative potentials [9]. Zhurkov et al. [10] stated that physical mechanisms and apparent characteristics of fatigue depend on the structure of materials and physical conditions, chemical composition, and the kind of utilized load. They modified the inhomogeneous field mechanism (IFM) model for the development of distinct fatigue degradation. In thermodynamic interpretation, the microscopic physical mechanisms are not well considered in this work. The existence of entropy generation is as such a prerequisite for the development of fatigue degradation for the explanation to understanding the microscopic physical mechanisms. 

Given that the fatigue process is usually accompanied by energy transformation, developing a thermodynamic framework to study its characterisation is reasonable. Energy dissipation is an irreversible phenomenon, which in turn depicts the theory of entropy as an ideal tool in studying the fatigue process [11]. Meneghetti [12] studied the theoretical framework and the equivalent experimental techniques to calculate the dissipated heat energy in a structural volume surrounding the crack tip. The dissipated heat energy in a unit volume of the material per cycle (Q) can be adopted as an index of fatigue damage [13], whereas the average heat energy per cycle Ǭ* can serve as an elastic–plastic fracture mechanics parameter [14]. The thermodynamics framework has been recently applied in mechanical fatigue [15,16,17,18], which is not unexpected because fatigue degradation is an irreversible process that gradually ages the system until failure by fracture. Additionally, fatigue is a dissipation process, in which the accumulation of disorder is basically related to the generation of entropy based on the second law of thermodynamics [15,19,20,21] Thermal methods were used to study the dynamic crack behaviour of materials through the thermal signal time domain [22,23,24,25]. Furthermore, a cumulative entropy generation can provide estimation when crack initiation commences [26]. 

The entropy production that progressively accumulates is associated with the degradation or aging of the technique used and destroys the device until failure. The relationship between degradation and entropy generation because of irreversible processes was established within the system [27]. Particularly, material damage in different conditions, such as mechanical cyclic load, can be evaluated using the amount of entropy generation during degradation [28]. The driving motivation for degradation-entropy generation (DEG) theorem is that entropy monotonically increases, and free energy monotonically decreases for every ordinary process. Particularly, the entropy-generating irreversible process is present in all aging phenomena. Following the entropy increase, manufactured components return to their natural conditions through degradation, their integrity is consequently degraded and they eventually become non-functional [29]. However, within the framework of fracture mechanics, the entropy produced from crack growth has not been thoroughly investigated.

FCG for realistic structures involves VAL that leads to load interaction effects, and the result of FCG rate is difficult to predict. The entropy generated during the fatigue failure process can serve as a measure of degradation. Therefore, the FCG model called the entropy generation of fatigue crack (EGFC) for steel based on DEG theorem is proposed. An effective evaluation method for the FCG rate by using DEG theorem is essential, especially when employing the FCG test under spectrum loading. This study aims to understand how the DEG theorem can predict the FCG rate of a specimen, especially under spectrum loadings. The outcomes of the FCG test experiments and the theory revealed that fatigue degradation and entropy generation was closely related and that this relationship should be considered when evaluating the FCG rate based on the DEG theorem. Moreover, the results contributed fundamental improvements to the studies related to FCG rate without the need for conventional techniques based on empirical models, thereby easing the FCG rate prediction and lessening the necessary tests. Consequently, the generalisation of the predictions for any kind of spectrum loading will be relatively straightforward. For instance, the FCG rate was directly predicted from evaluation of the fatigue DEG. The advantage of using this method is the possibility of FCG prediction under spectrum loading. This step can be achieved through the measurement of the degradation coefficient of a given material under constant amplitude loading (CAL) by applying the concept that the total entropy generation is not dependent on load. The concept of DEG theorem is also reliable to evaluate the FCG rate.

## 2. Theoretical Model Based on Entropy Generation

### 2.1. Entropy Generation in an Irreversible Process

In an irreversible process, heat energy loss or dissipation can be observed because of intermolecular collisions and friction, which does not allow the recovery of energy when the process is reversed. Entropy can be defined as the irreversibility degree of a process. Sufficient amount of functional energy is lost due to dissipation or friction, which is disadvantageous to combustion reactions. Entropy is a non-existent form of energy that performs a beneficial work for the thermodynamic process. In conventional thermodynamics, reversible process entropy was described by Clausius as the ratio of heat energy transferred from the system to the absolute temperature. The equations of classical thermodynamics are not applicable for irreversible processes. The second law of thermodynamics could represent an inequality by introducing appropriate terms to account for the entropy’s irreversible production. This inequality can be expressed as

(1) $$\Delta \gamma =\sum_{m}\gamma_{m}+\sum_{n}\frac{Q_{n}}{T_{n}}+\Delta \gamma_{irr}$$

where Δγ is the rise in the entropy of the system, $\sum_{m}\gamma_{m}$ is the net sum of the entropies transmitted into the system through transfer of matter, $\sum_{n}\frac{Q_{n}}{T_{n}}$ is net sum of the entropies transmitted into the system through heat transfer, *Q* is the transferred heat energy transferred, *T* is the absolute temperature and $\Delta \gamma_{irr}$ is the entropy generated by the irreversible processes happening within the system. The system endures an irreversible process and raises the entropy. The alteration in entropy caused by the irreversible processes that happens inside the system, which is always positive regardless if no matter or heat energy is transmitted into or out of the system. A simple example is the energy degradation utilised for mechanical works, wherein the dissipation of the internal energy occurs in the material body. If the dissipated energy *dE_d_* have the same impact as that of the absorption *dQ*, which is in the form of heat, because of a constant equilibrium temperature *T*, then the entropy production rate can be presented as [30]

(2) $${\left(\frac{d\gamma }{dt}\right)}_{irr}=\frac{1}{T}\frac{dE_{d}}{dt}$$

where ${\left(\frac{d\gamma }{dt}\right)}_{irr}$ and $\frac{dE_{d}}{dt}$ are the irreversible entropy production and energy dissipation rates, respectively.

Naderi and Khonsari [31] stated that entropy is produced by the plastic work divided by temperature. The plastic work is mostly (around 90% for steels) dispersed into heat, and the remaining tiny portion in the material partakes in microstructural evolution [32]. The measurement of entropy production aims to assess the material damage. Part of the plastic work that dissipates through heat to the environment does not influence the degradation and damage and thus should be neglected when measuring the entropy generation during plastic deformation. In this study, the phrase ‘dissipated energy’ (*E_d_*) is used instead of the plastic work *W_p_* in computing the entropy production.

The rise in entropy is caused by the alteration in internal energy (i.e., the total energy in the thermodynamic structure during solids’ deformation). Internal energy involves the strain energy absorbed during heat generation, the plastic work under strain hardening, the deformed solid and the sound released because of cracking [33]. The total energy can be obtained from the area under the load deformation curve of a solid. The total energy without the elastic strain energy renders the dispersed energy, which is entirely recoverable. This dissipated energy divided by the temperature is the entropy produced in the absence of heat transfer. There is a rise in stresses until reaching the strength limit on loading a material. When the stress reaches the tensile strength at a point, the material might crack. Entropy is generated by the solid that has a growing crack despite exhibiting an elastic mechanical response, signifying that irreversible crack propagation is an irreversible thermodynamic process [34].

### 2.2. Entropy Generated during FCG

FCG is an irreversible procedure that is related to the increase in entropy according to the second law of thermodynamics. The rate of entropy generation stated by Equation (2) with regards to the number of load cycles, which is relevant to fatigues, can be rewritten as

(3) $$\left(\frac{d\gamma_{irr}}{dN}\right)=\frac{1}{T}\frac{dE_{d}}{dN}$$

where $\gamma_{irr}$ it the irreversible entropy, *E_d_* represents the dissipated energy, *T* is the absolute temperature (i.e., the surface temperature of specimen) and *N* is the number of loading cycles. Hence, the total entropy can be computed by integrating Equation (3) from 0 until the point of fracture *N_f_*.

(4) $$\gamma_{f}=\int_{0}^{N_{f}}\left(\frac{E_{d}}{T}\right)dN$$

where $\gamma_{f}$ is the total entropy production of a fatigue failure.

The entropy pertaining of a thermodynamic system that undergoes cracking involves all the energy lost either because of the propagation or crack formation or through other dissipative mechanisms. For ductile materials (e.g., metals), energy is dissipated when a new crack surface is formed or when the plastic zone that lies ahead of the crack tip is lost. In this case, plasticity is the dominant dissipative mechanism.

## 3. Methodology

### 3.1. Entropy Generation of the EGFC Model Development Using DEG Theorem

Bryant et al. [27] introduced the DEG theorem, in which only one dissipative process *p* is responsible for the degradation of a system that generates entropy. According to the theorem, the crack length *w* indicates a measure of the degradation of the system that is dependent on the dissipative process, where *w* = *w* (*p*). Similar to entropy generation, the degradation rate *D* = *dw*/*dt* can be obtained by employing the chain rule.

(5) $$D=\frac{dw}{dt}=\left(\frac{\partial w}{\partial p}\frac{\partial p}{\partial \zeta }\right)\frac{\partial \zeta }{\partial t}=YJ$$

where $Y=\frac{\partial w}{\partial p}\frac{\partial p}{\partial \zeta }$ is the degradation force. The degradation of a given system is dependent on the equivalent *p* as the representative of entropy generation. Given that thermodynamic flow *J* is probably the common parameter in Equation (5), the degradation coefficient can be obtained as:

(6) $$B=\frac{Y}{X}=\frac{\left(\partial w/\partial p)(\partial p/\partial \zeta \right)}{\left(\partial_{i}S/\partial p)(\partial p/\partial \zeta \right)}=\frac{\partial w}{\partial_{i}S}|$$

where *B* describes the interaction of the degradation and entropy generation with *p*.

The crack length is considered as *w*, where *w* = *a* in Equation (5). Therefore, degradation can be denoted as $a=a\left\{W_{p}\left(N\right)\right\}$, where *W_p_* is the plastic energy generation at the tip of the crack and is probably the main dissipative process with the number of cycles *N* as the phenomenological variable. Numerous studies have attempted to the plastic energy generation *W_p_* as a function of the FCG rate [35,36,37]. According to Equation (5), it can be written as dadt=YJ, where J=dNdt and Y=(dadWp)x(dWpdN). With the assumption that the plastic energy is dissipated as entropy generation, $dW_{p}=Td_{i}S$. Hence, the entropy generation can be defined as

(7) $$\gamma =\frac{d_{i}S}{dt}=\frac{\partial_{i}S}{\partial p}\frac{\partial p}{\partial N}\frac{\partial N}{\partial t}=\frac{f}{T}\frac{dW_{p}}{dN}$$

where *f* is the frequency of the test and *T* is the surface temperature. By substituting $X=\left(1/T\right)\left(dW_{p}/dN\right)$ into Equation (5), and following expression was obtained.

(8) $$D=\frac{da}{dt}=YJ=BXJ=B\frac{f}{T}\frac{dW_{p}}{dN}$$

As previously mentioned, *B* in Equation (8) measures how crack growth and entropy generation interrelate on the dissipative level of the plastic deformation process. The rate of crack growth given with respect to the number of load cycles can be expressed as

(9) $$\frac{da}{dN}=\frac{da}{fdt}=\frac{B}{T}\frac{dW_{p}}{dN}$$

In terms of energy balance, the total energy needed to propagate a crack with a unit distance in a specific material is independent of the energy dissipation mechanism. Therefore, the energy absorbed per unit growth of the crack is equal to the dissipated plastic energy of the cyclic plastic zone per cycle [38]. This concept can be mathematically expressed as

(10) $$W_{c}\delta a=\frac{dW_{p}}{dN}$$

where *W_c_* is the plastic dissipation energy until fracture. By replacing the value of *W_c_*, the crack growth rate *da*/*dN* is obtained as

(11) $$\delta a=\frac{da}{dN}=\frac{1}{W_{c}}\frac{dW_{p}}{dN}$$

Combining Equations (9) and (11), we obtain

(12) $$\frac{da}{dN}=\frac{1}{W_{c}}\frac{T}{B}$$

Several methods have been developed to assess *W_c_*. In this study, the FCG rate was controlled using the crack tip opening displacement (CTOD). Dependency on ∆*K*^2^ can be obtained, and the theories based on crack opening displacement will result to the Paris law exponent *m* = 2 [39]. Therefore, a correlation introduced by Skelton et al. [40] was employed.

(13) $$\frac{da}{dN}=\frac{\Delta K^{2}\left(1-\nu \right)}{2\pi EW_{c}}$$

where ∆*K* is the stress intensity factor, ν is the Poisson’s ratio and *E* is Young’s modulus. The EGFC model can be obtained by substituting Equation (12) into Equation (13).

(14) $$\frac{da}{dN}=B\frac{1}{T}\frac{\Delta K^{2}\left(1-\nu \right)}{2\pi E}$$

Equation (14) represents the Paris–Erdagon law of crack growth, where the constant *C* is expressed as:

(15) $$C=B\frac{1}{T}\frac{\left(1-\nu \right)}{2\pi E}$$

Equation (15) indicates the relationship between *C* and *B*, which is associated with entropy generation.

### 3.2. Prediction of Fatigue Life

In this study, the total fatigue life of each FCG test was estimated by integrating the Equation (14) using Simpson’s rule [41]. Three neighbouring crack lengths, namely, *a_j_*, *a_j+1_* and *a_j+2_* were used to calculate the number of cycles based on Simpson’s rule. The number of cycles for crack length to propagate from distance *a_j_* to *a_j+2_*, can be obtained as:

(16) $$\Delta N_{j+2}=\int_{a_{j}}^{a_{j+2}}\left[y\right]da=\frac{a_{j}\left(r^{2}-1\right)}{6r}\left[y_{j}r\left(2-r\right)+y_{j+1}{\left(r+1\right)}^{2}+y_{j+2}\left(2r-1\right)\right]$$

where *j* is the number sequence, *y_j_* is the difference between the numbers of cycles for crack length interval, *r* is the interval between the crack length and *y* represents the *dN*/*da* in Equation (14).

(17) $$y=\frac{dN}{da}=T\frac{1}{B}\frac{2\pi E}{\Delta K^{2}\left(1-\nu \right)}$$

### 3.3. Materials and Specimens Preparation

In this study, a low carbon steel sample was used because it has the largest temperature range within the Eutectoid temperatures, minimum temperature of austenite A_1_ and lower-bound temperature for austenite A_3_ in the iron–carbon phase diagram amongst other types of carbon steel. A dual-phase steel was synthesised by subjecting low carbon steel samples to inter-critical annealing from a temperature above A_1_ but below A_3_ between the two phases’ (i.e., ferrite + austenite) regions for a given period, followed by water quenching. The chemical composition of this steel is shown in Table 1. Two successive heat treatment processes were conducted to obtain the dual-phase material. In the first process, the as-received specimens were annealed at 760 °C for 90 min, followed by water quenching (inter-critically annealed) to achieve the martensite phase. The temperature range was selected on the basis of the highest fatigue strength [42]. The second process involves tempering at 400 °C for 2 h and then cooling at room temperature to eliminate the residual stresses and improve the toughness. This tempering temperature was chosen because low carbon steel is tempered after heat treatment between 200 °C and 600 °C [43,44,45]. All specimens (as-received and dual-phase specimens) were mechanically polished to remove all damaged layers.

### 3.4. Tensile and FCG Rate Tests

**Figure 1:**
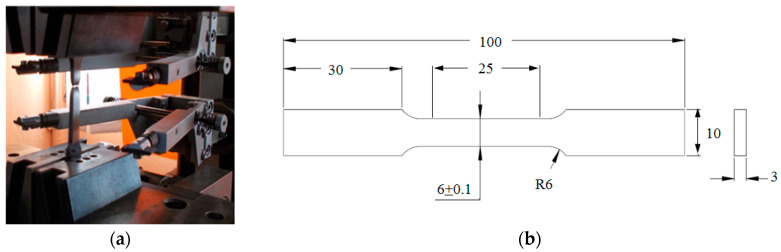
Tensile test; (**a**) experimental setup, (**b**) specimens’ geometric dimensions (mm).

The tensile specimens were prepared according to ASTM E8 in sub-size dimensions with a gauge length of 25 mm (Figure 1). Tensile tests were also conducted according to ASTM E8 procedures at room temperature using a universal testing machine with a cross-head speed of 1.8 mm/min (equivalent to a strain rate of 0.001 s^−1^) to investigate the mechanical properties of the dual-phase steel samples.

**Figure 2:**
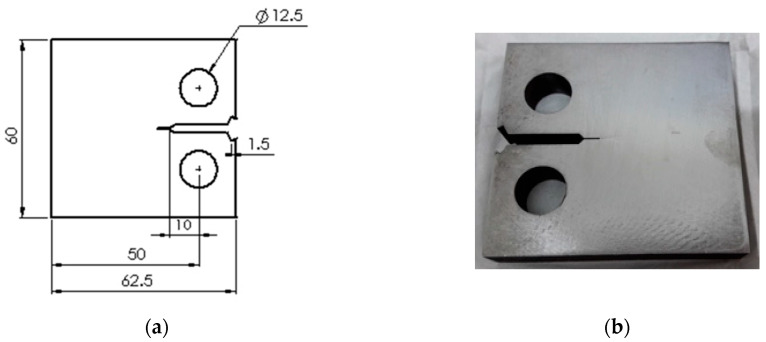
Compact tension (CT) specimen for fatigue crack growth (FCG) test; (**a**) specimens’ geometric dimensions (mm), (**b**) actual machined specimen.

The geometric dimensions of the compact tension (CT) specimens were set according to ASTM E647, where thickness (B) = 12 mm and width (W) = 50 mm (Figure 2). Wire cut electric discharge machining (EDM) was used to cut the specimens. The residual compressive stresses from the milling process [46] were reduced by cutting a 4 mm sharp notch using EDM.

**Figure 3:**
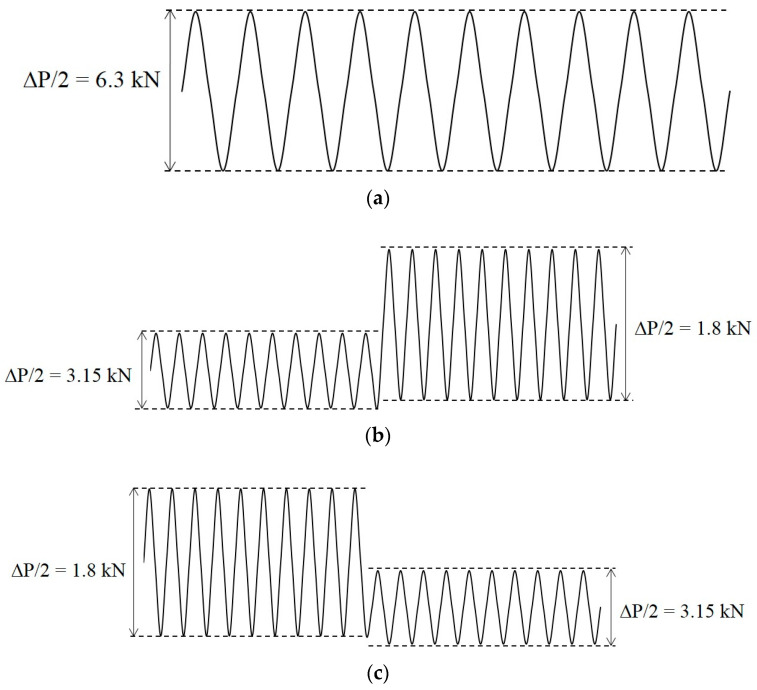
Schematic of the applied two-stage block loading sequences: (**a**) constant amplitude loading (CAL); (**b**) L-H; (**c**) H-L.

The FCG tests were performed according to ASTM E647 procedures by utilising a servo-hydraulic universal test with a 100-kN capacity load cell. All experiments were conducted in an ambient setting with a load ratio R = 0.1 and loading frequency of 10 Hz sinusoidal waveform [47,48,49]. Three additional experimental tests were executed to represent the three types of loading. The FCG test classification was divided into three types: CAL, VAL and a two-step sequence loading involving high–low (H-L) and low–high (L-H) loadings. R was maintained constant throughout the experiment (Figure 3). In the two-step H–L loading experiment, the first loading step casted a considerable impact on the subsequent crack growth (i.e., the second loading step) when either R or the minimum load is equal in both steps [50].

The FCG tests commences with a fatigue pre-cracking process that allowed the formation of a crack via the sharp notch, which represents each CT specimen. The fatigue pre-cracking was conducted to produce the sharpened fatigue crack with sufficient straightness and size. The value of *K*_max_ was set as 32 MPa.m^1/2^, along with the sinusoidal cyclic loading with R = 0.1 and a 10-Hz frequency to allow the desired crack to appear. The pre-crack value will surpass 0.10B almost all the time, adopting the value of the thickness *h* or 1.0 mm (0.040 in.), whichever is greater. The crack size on both sides (back and front) of the specimen was measured to ensure that the crack symmetry is maintained as indicated in the ASTM standard. The pre-crack length was set as 0.10B (1.2 mm) in this study. All specimens were then subjected to the FCG test under VAL and CAL conditions until fracture.

All the CT specimens were subjected to mode I opening loading. The compliance method with CTOD was applied to measure the fatigue crack length by employing a clip gauge at the notch mouth.

(18) $$\alpha =a/W=1.0010-4.6695u_{x}+18.46u_{x}{}^{2}-236.82u_{x}{}^{3}+1214.9u_{x}{}^{4}-2143.6u_{x}{}^{5}$$

(19) $$u_{x}={\left\{{\left[\frac{EVB}{P}\right]}^{1/2}+1\right\}}^{-1}$$

where *a* is the crack length, *W* is the specimen width, *B* is the specimen thickness, *E* is Young’s modulus and *V* is the CTOD. ∆*K* was calculated as

(20) ΔK=ΔPBW(2+α)(1−α)32(0.886+4.64α−13.32α2+14.72α3−5.6α4)

where ∆*P* is the applied load range and *α* is the relative crack length (*a*/*W*).

### 3.5. Temperature Measurement

**Figure 4:**
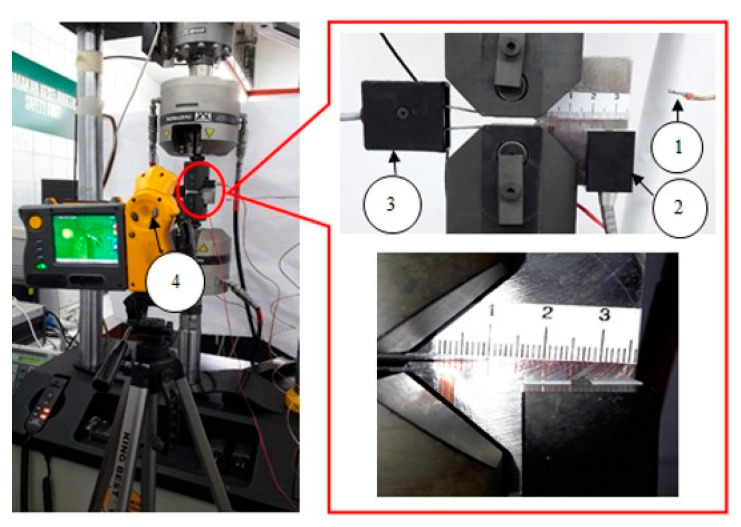
Schematic for FCG (fatigue crack growth) rate test: (1) Thermocouple-measured ambient temperature; (2) thermocouple-measured surface temperature; (3) clip gauge, (4) high-resolution IR thermal imager.

**Figure 5:**
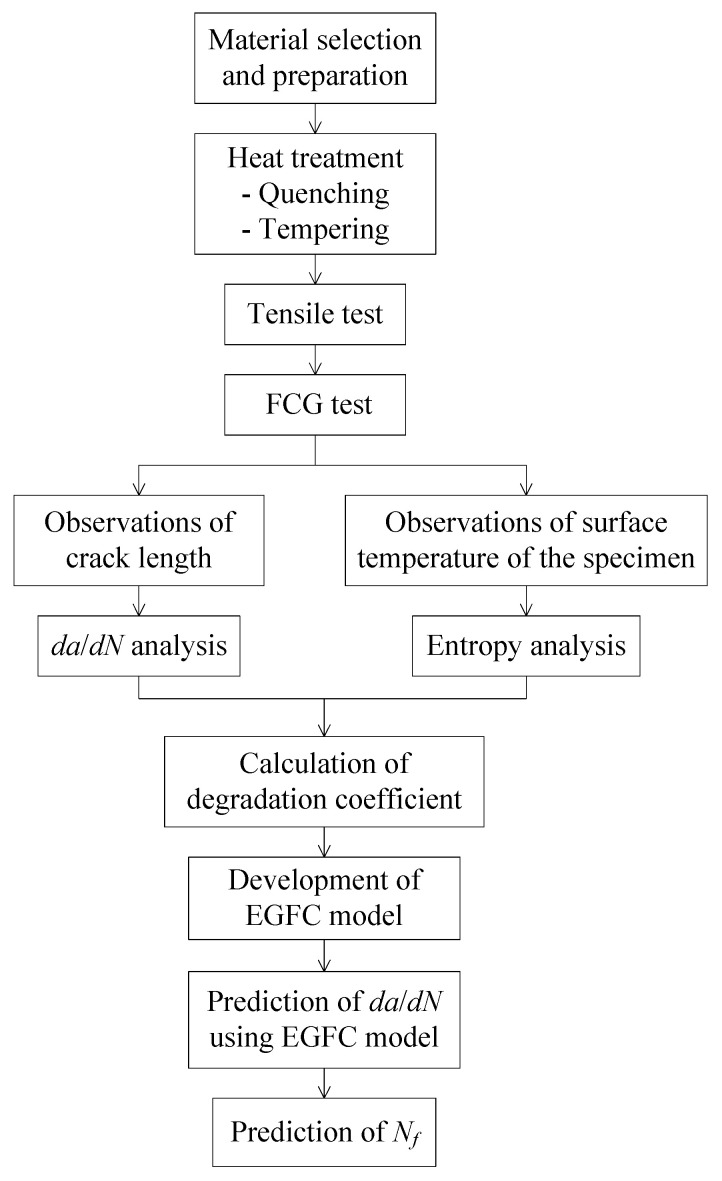
Flow of FCG rate prediction.

Two thermocouples were used to determine the temperature evolution in the test samples. Naderi and Khonsari [51] utilised these thermocouples to oversee the specimen’s thermal response. The delamination of the thermocouples was mitigated using magnetic thermocouples to ensure that good contact is maintained during the initiation at the specimen surface. In the FCG test, the surface temperature evolution of the specimen with respect to the crack growth was recorded using a magnetic thermocouple. The second thermocouple was positioned near the specimen to record the ambient temperature. This step ensures that no temperature change will occur in the surface of the specimen and that the ambient temperature will not exert great influence. A data acquisition tool was utilised to gather thermocouple data. Given that positioning the thermocouple at the spot where the crack occurs without disturbing the crack movement is difficult, a high-speed high-resolution infrared (IR) thermal imager was deployed to calibrate the thermocouples and minimise the error in the computation of entropy production, particularly at the start of the test, and to capture the crack growth’s thermal image. The temperature was at the crack tip. Figure 4 and Figure 5 illustrates the experimental setup of the FCG test for temperature measurement and the experimental process flow, respectively.

## 4. Results and Discussion

Experimental results are presented to determine the validity of the model in predicting the FCG rate. The proposed model can be useful in producing accurate predictive results of the measured FCG rate under spectrum loading.

### 4.1. Mechanical Properties

The heat treatment caused a more effective change in the mechanical property of the dual-phase steels than in the as-received steels. Table 2 presents the differences in the mechanical property of the as-received and dual-phase steel specimens. The ductility values of the latter were lower than those of the former. This result can be attributed to the existence of harder and less ductile ferrite matrix in the microstructure of the dual-phase steel specimen compared with the as-received steels. The as-received specimens had high ductility because of their softer ferrite–pearlite structures compared with the martensitic structures in the dual-phase steel [52].

The mechanical properties significantly changed after the heat treatment as proven by the increase in yield strength and ultimate tensile strength of the dual-phase steels. Both properties rapidly increased after heat treatment, reaching 495 and 597 MPa for the dual-phase and as-received steels, respectively. This finding specifies that the strength values of the dual-phase steels were higher than those of the as-received steel because of the presence of the harder second phase in the former [53]. The strength of the martensite produced in the soft ferrite matrix might differ from the structure formed when the steel was changed from austenite to 100% martensite [54,55].

### 4.2. FCG under Cyclic Loading

**Figure 6:**
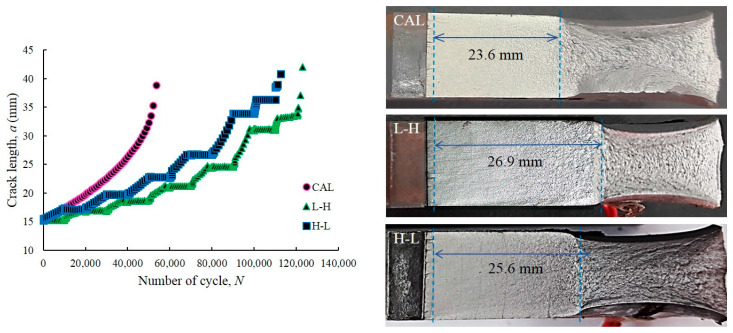
Fatigue life of dual-phase steels based on crack length observations.

The FCG process is often classified into three stages: slow (stage I), stable (stage II) and rapidly growing regions (stage III). Figure 6 presents the fatigue life of the as-received and dual-phase steels based on crack length measurement during the FCG test. The figure shows that the crack initially grew at a slow rate and started to accelerate when the crack length increased after many cycles. The final point for each curve is the final fracture during the FCG testing. On the one hand, the analysis of FCG for VAL condition under L-H loading endured the longest life at 123,000 cycles, whereas that for CAL condition had the shortest life at 53,674 cycles. On the other hand, the analysis of FCG for VAL condition under H-L had a fatigue life of 112,683 cycles, which is within the range of the two analyses. The total cycles until failure depended on the type of the applied load. For high stresses, the crack growth rate represented by the slope of the curve was high at a given crack length and the FCG life (i.e., total number of applied cycles) was short. The highest stress level was observed at the shortest crack length during fracture. The magnitude of the applied stress and the fracture resistance of the material influenced the life until fracture of a given initial crack size.

The fatigue life under VAL conditions in both loadings was longer than that under CAL conditions because of the interaction of overload and underload cycle loadings. The sequence loading enhanced the crack growth retardation, which was simplified. This trend is similar to the findings of other research [56,57,58], wherein a slower crack growth was observed under low to high sequence loading compared with high-to-low sequence loading. Great crack growth retardation signifies long sample life. Other research suggested that the level and sequence of the load cycles can cause retardation or acceleration of crack growth, which can influence the fatigue life [59]. The retardation of crack growth is related to the changes in the size of plastic zone at the crack tip [60,61,62]. Other researchers proved that large plastic zones exert great effect on FCG retardation [63]. The crack growth in the large plastic zone caused the retardation of FCG rates. When the crack grew out of the overload plastic zone, a normal crack growth rate was observed upon achieving the original size of the plastic zone. This phenomenon is due to the increase in the material resistance passing through the plastic zone, whereas the overload is due to retardation of the FCG rate.

### 4.3. Temperature Evolution for Cyclic Loadings

**Figure 7:**
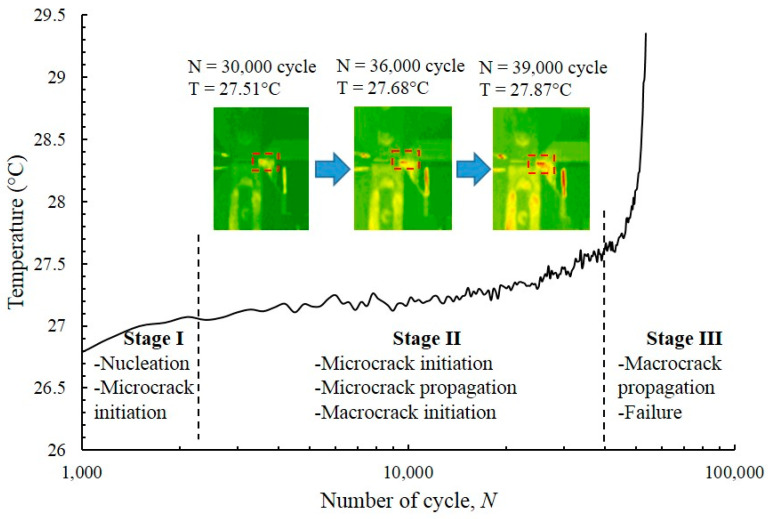
Typical temperature evolution during CAL.

The representative entropy accumulation of the dual-phase specimen was plotted as a function of the number of cycles in the FCG test under CAL condition (Figure 7). The plot shows that the evolution of temperature went through three distinct stages [64]. In the first stage, the surface temperature rapidly rose at the start of the test because of the reaction of the material to any unexpected movement, defect and dislocation caused by surface intrusion and extrusion. The temperature stabilised in the second stage, and then abruptly increased before the occurrence of failure, which denotes the third stage.

In the first stage, limited temperature evolution implies a minimal number of cycles, which is ≈10% of the specimen’s lifespan. [65] In this stage, surface temperature increased with energy density because of hysteresis impact and the accelerated heat generation versus heat loss caused by the specimen via radiation and convection. Moreover, a significant rise in temperature occurs because of the enhanced energy release because of the crack initiation events in the localised plastic zone, which increased the plastic zone’s size at the notch tip. In the second stage, the response of the specimen towards cyclic stress, along with strain stability, resulted in the balance between energy dissipation and generation, which facilitated stable temperature readings. During this stage, a slight decrease in temperature was observed as new surfaces formed because of the generation of microcracks, which leads to the heat loss in the surroundings [66]. Finally, the third stage took up around 5–10% of the entire fatigue life. A sudden rise in temperature signifies a small number of cycles, which depicted rapid crack propagation prior to failure. The failure resulted in a large plastic deformation near the macrocrack tip, which was generated because of the stress concentrations near the cracked tips [67].

**Figure 8:**
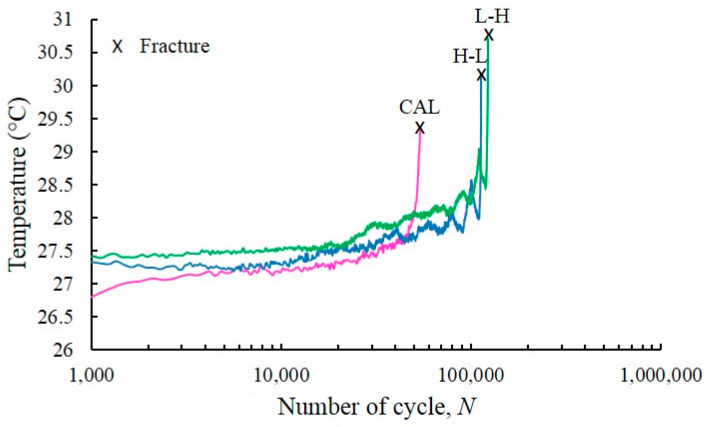
Temperature evolution during FCG test under spectrum loading conditions.

Figure 8 demonstrates the disparity in temperature evolution under VAL and CAL conditions, where H-L and L-H loading were observed during the entire FCG test. The pattern of temperature evolution under the both conditions was similar, and the evolution process comprises three distinctive phases. With the application of the load, a fluctuation of the temperature evolution was observed under VAL, which included L-H and H-L loadings. However, irrespective of the fluctuation, the mean temperature still increased. The temperature oscillation was present during each fatigue cycle in the entire FCG test because of the thermoelastic effect. Figure 8 shows that the temperature fluctuations during the course of the FCG test until fracture occurs under VAL and CAL conditions were not significantly high and were approximately 3 °C. The temperature change percentage was below 1% (i.e., $\frac{\Delta Q}{Q_{0}}\approx 1\%$).

**Figure 9:**
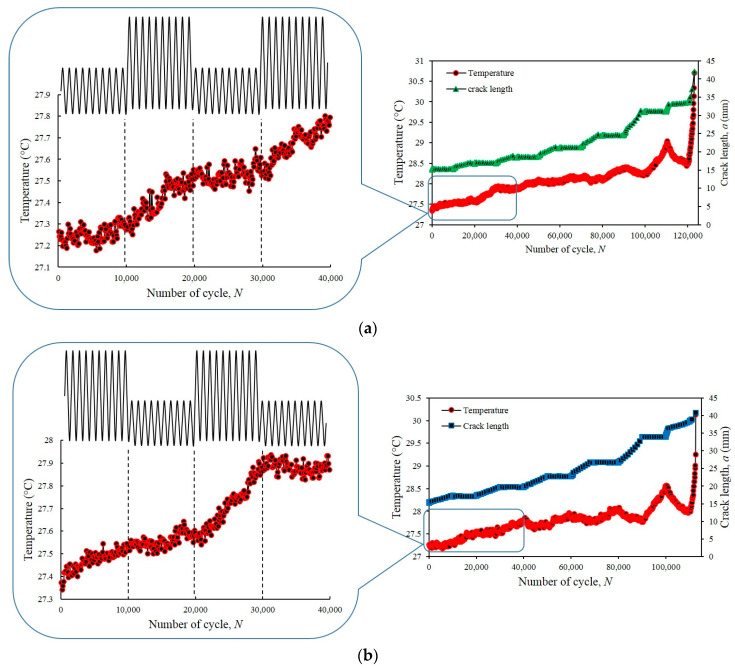
Temperature profile and crack length during the initial FCG test under sequence loading: (**a**) L-H; (**b**) H-L.

Figure 9 shows the temperature profiles at the initiation phase of the FCG test under VAL condition, which involve two steps of L-H and H-L. The mean temperature increased in proportion to the applied load applied under the condition when the load started to fluctuate in a sinusoidal wave. An increase in the temperature evolution was observed as the applied load increased when the load changed from L-H. A similar phenomenon was observed when the load changed from H-L, in which the temperature evolution showed a downward trend with the decrease in the applied load. The temperature evolution was affected by the changes in load application because the heat dissipation was dependent on the amount of load applied to the specimen. The temperature evolution increased as the applied load increased because the stress became concentrated at the crack tip [67].

### 4.4. Entropy Generation

**Figure 10:**
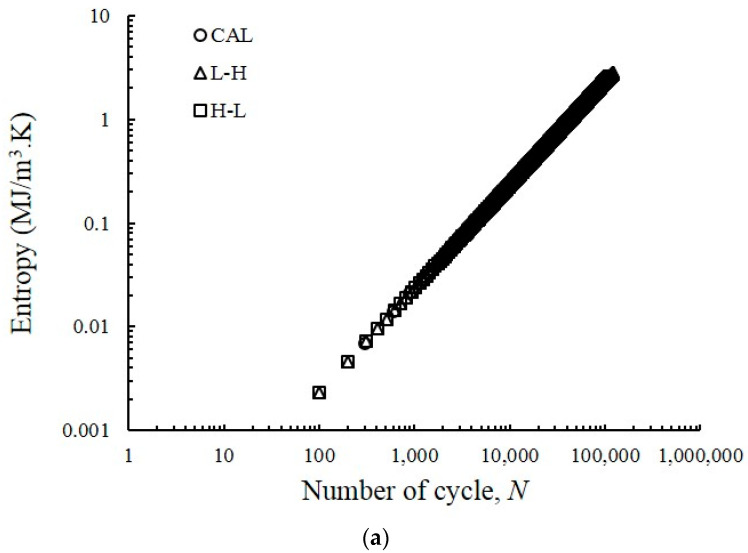

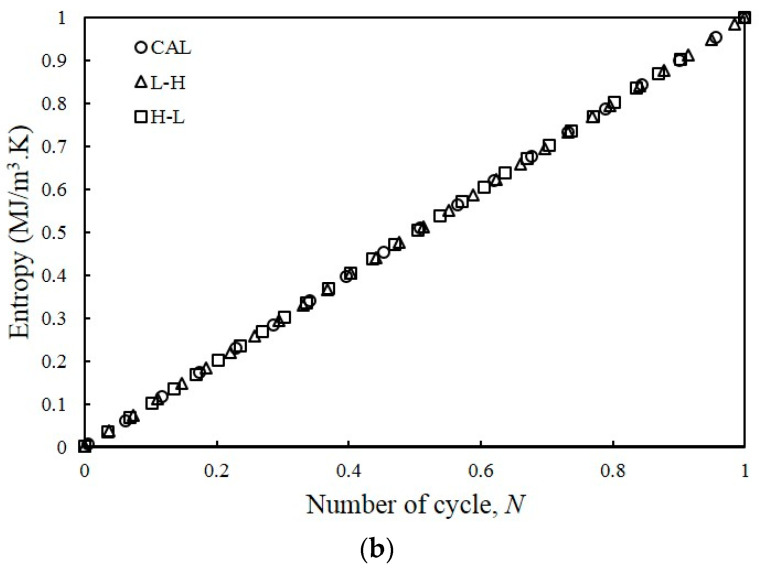
(**a**) Evolution of the total entropy generation in the FCG test under spectrum loading conditions; (**b**) normalised entropy generation.

To determine the evolution of entropy generation, the complete FCG test was calculated. The equation for entropy generation was integrated at beginning of the test throughout a number of cycles until the fracture appeared. The outcomes of the overall entropy generation under VAL and CAL conditions are plotted in Figure 10. During the initiation of the test, the overall entropy generation was zero. However, upon reaching the fracture point, linear progression was observed. The overall entropy generation was 1.24, 2.84 and 2.61 MJ/m^3^.K for dual-phase steel under CAL, L-H and H-L conditions, respectively. The variation in the number of cycles until failure with varying load application is the main reason behind the difference in the values of the total entropy generation under VAL and CAL conditions. The results indicate the entropy generation increases as the number of cycles until failure increases. The accretion of entropy generation was low when the load amplitudes were high (i.e., small number of cycles until failure) but increased when the load amplitude decreased.

According to the findings of other researchers, the total entropy generation of a particular material was not influenced by the load, frequency, or geometry; this value is constant during failure [28,31]. For materials with different characteristics, an entirely different total entropy generation at the fracture point can be expected. For example, stainless steel 304 or aluminium 6061 exhibits an overall entropy generation within the ranges of 60 and 4 MJ/m^3^.K, respectively. Additionally, the increase in entropy at the point of failure can be considered as a characteristic of the material itself [31]. The results presented in Figure 10 differed from the previous studies on the entropy of fatigue life [68]. Other researchers reported a constant entropy generation at the end of the fracture. This difference lies in the energy dissipation calculation. In the present study, energy dissipation was determined by calculating the area under the load deformation curve minus the elastic strain energy. Naderi et al. [68] used the plastic strain energy density, which was calculated using the relationship presented by Morrow for the fully-reversed non-notched plastic strain that dominated the loading conditions.

This research presented the approximately linear slope of the total entropy generation that was plotted as a function of the number of cycles until failure of the material under CAL and VAL conditions. This observation revealed a monotonically rise in entropy generation until the failure point under both conditions. A methodology for the prediction of FCG rate for a given material under CAL and VAL conditions was developed through the linear relationship between entropy generation and fatigue failure. Furthermore, the prediction of FCG rate under VAL was difficult and complex. Therefore, a simple method was developed to predict the FCG rate of the specimen under both conditions using the CAL condition.

### 4.5. Relationship between Degradation Coefficient and Entropy Generation

**Figure 11:**
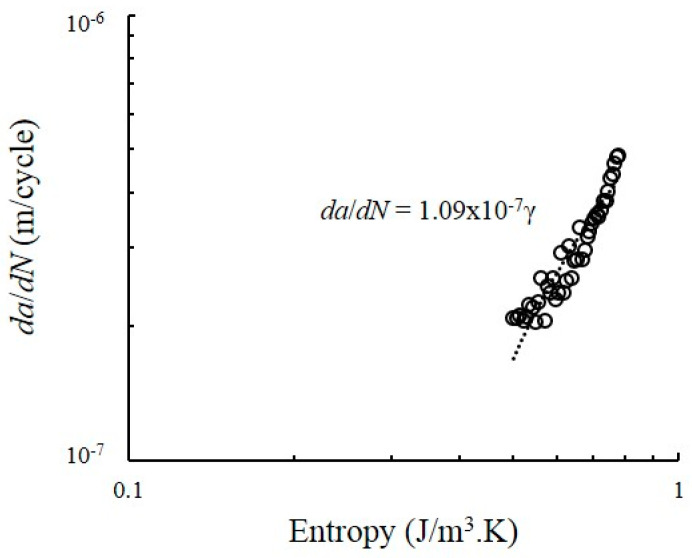
Relationship between degradation parameter rate and entropy generation under CAL condition.

The relationship between the degradation rate and the entropy generation rate associated with *B* for dual-phase steels is shown in Figure 11. The degradation rate, which in this case is the crack length *da*/*dN* demonstrate a linear relationship with the components of entropy generation, that is, dadN=Bγ. The value of *B* for dual-phase steel under CAL condition is 1.09 × 10^−7^. This value is used to predict the FCG rate for dual-phase steel under CAL and VAL conditions, determine the interaction between crack growth and entropy generation towards the degree of dissipative plastic deformation process and predict the FCG rate based on the DEG theorem.

### 4.6. FCG Prediction

**Figure 12:**
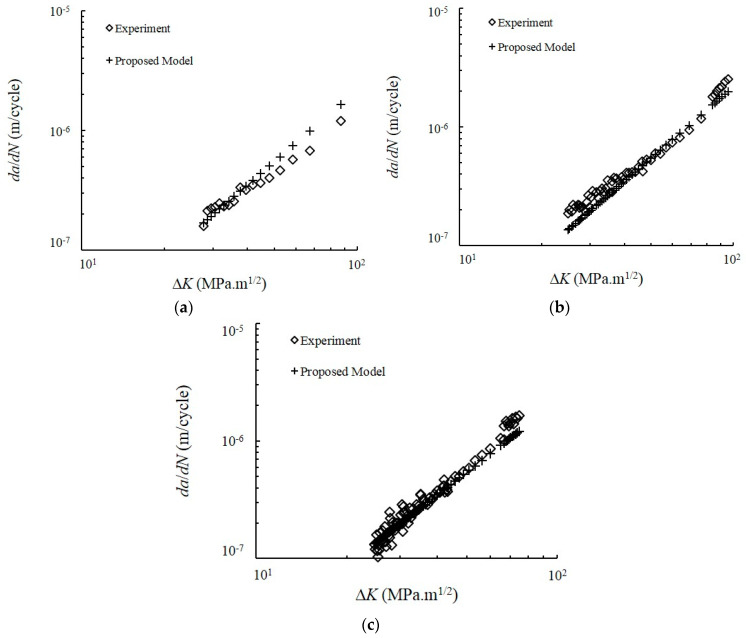
Comparison of the predicted crack growth rate using entropy generation of fatigue crack (EGFC) model with experimental data under spectrum loading conditions: (**a**) CAL; (**b**) L-H; (**c**) H-L.

**Figure 13:**
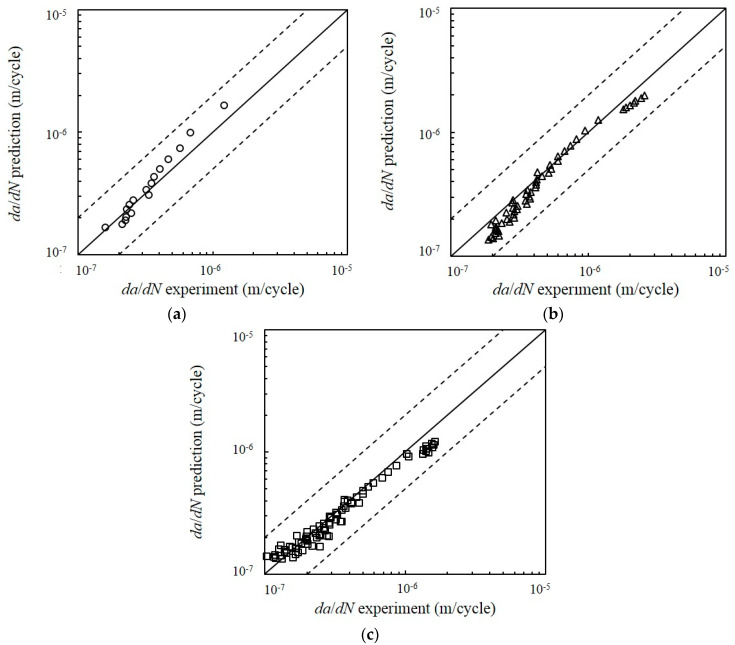
Comparison of the predicted FCG rate using EGFC model with experimental data using conventional scatter band for each type of load: (**a**) CAL; (**b**) L-H; (**c**) H-L.

The EGFC model was compared with the measured Paris regime crack growth data for dual-phase materials. The comparison between *da*/*dN* and the ∆*K* of the experimental data is represented by Equation (15) (Figure 12). The results showed a strong agreement between the EGFC model and the measured crack growth data. A visual illustration of the FCG rate is given in Figure 13 using the conventional scatter band. The FCG rate points were scattered around the 1:1 correlation line and was limited in the safety factor band (dashed line) of the 1:2 and 2:1 correlation lines. The scatter band showed the variations in the FCG rate prediction values for each type of loading. All data points for all types of loading fell within the range of the plotted accuracy band. Furthermore, all data points were considered accurate because of their proximity to the 1:1 correlation line. This finding shows that most of the predicted FCG rates are close to the experimental values. The results revealed an acceptable correlation between the entropy generation and the FCG rate under CAL and VAL conditions.

The accuracy of the EGFC model was compared with that of the experimental data determined by a statistical test of the variance of the given residuals. The RMSE values of the as-received and dual-phase steels are presented in Table 3. The RMSE values of the latter under CAL, L-H and H-L are 1.0291 × 10^−7^, 1.9769 × 10^−7^ and 1.5409 × 10^−7^ m/cycle, respectively. This finding indicates that the EGFC model produced an accurate prediction of the FCG rate under CAL and VAL conditions and the experimental data for the measured crack growth had proximal scales with ∆*K*^2^. The EGFC model was developed to predict the FCG for materials with an *m* value that is approximately or equal to two. Given that the FCG rate in this study was controlled by the CTOD, a dependency on ∆*K*^2^ was observed [39], and a value of *m* = 2 was obtained from the theories based on crack opening displacement [40]. The predicted results were in good agreement when the measured data at the mid-range values of ∆*K* are used. Although the predicted slope had errors, the Paris regime data throughout the log-og plot was accurately represented by the proposed model. In conclusion, the EGFC model is applicable for CAL and VAL conditions. The minor errors can be attributed to the complete coupled thermomechanical solution of the problem, which was excluded from this study but will be investigated in the future.

The coefficient of variations (CVs) of the measured FCG rate data were calculated and compared using traditional techniques based on the experiments and on the DEG theorem to further explore the effects of FCG rate prediction using entropy generation. The CV is a relative dispersion measure that describes the standard deviation as a percentage of the arithmetic mean of a set of observations [69].

(21) $$CV=\frac{\sigma }{\mu }\times 100\%$$

where *σ* and *μ* are the FCG rate’s standard deviation and arithmetic mean, respectively. When CV = 0, all values are the same regardless of the variability or uncertainty. High CV value indicates great data transmission. Table 4 presents the values of the CVs for FCG rates under different types of loading. Under all loading conditions, the CV of the FCG rate based on the DEG theorem was higher than those obtained using traditional techniques. In conclusion, entropy generation is sensitive to variations when predicting the FCG rate.

### 4.7. Fatigue Life Prediction

**Figure 14:**
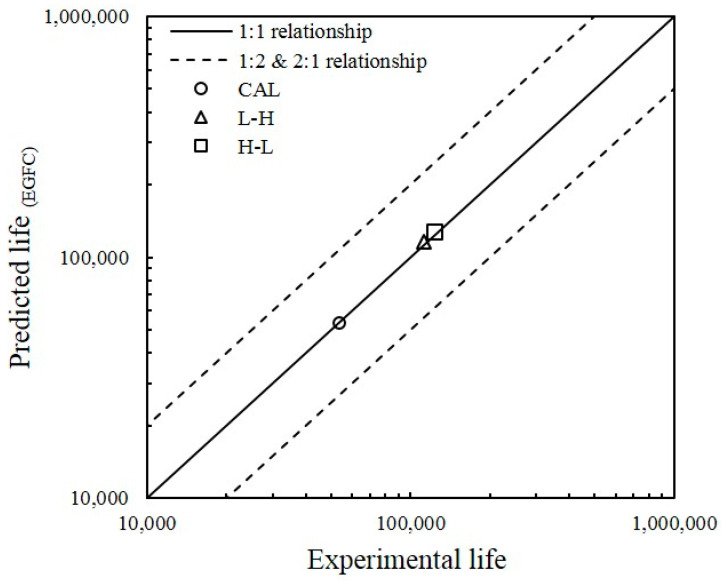
Correlation between the predicted and experimental results under different types of loads.

Figure 14 shows the correlation between fatigue life estimation and experimental outcomes. The fatigue life points scattered around the 1:1 correlation line are limited in the safety factor band of the 1:2 and 2:1 correlation lines. Results with a scattering factor of 2 on lifetimes are usually accepted in fatigue life analysis [70]. The finding shows that most of the fatigue life obtained from the experimental work are near to the predicted value. The plot indicates that the predicted fatigue life is in agreement with the actual fatigue lifespan of the specimens.

The accuracy of the predicted fatigue life can be obtained as

(22) $$\delta \%=\frac{N_{pre.}-N_{exp.}}{N_{exp.}}\times 100\%$$

where *N_pre._* is the predicted life and *N_exp._* is the experimental life, which represents the specimen’s number of cycles until failure during the FCG test. Table 5 presents the error values of the predicted and experimental data under CAL and VAL conditions. The results verifies that the predicted fatigue life is in good agreement with the experimental data under CAL and VAL conditions; the error is less than 5%. Moreover, the observed correlation between the FCG rate and the entropy generation is acceptable for the evaluation of the FCG rate based on DEG theorem when the FCG tests are conducted under CAL and VAL conditions. Therefore, even if the total entropy generation under CAL and VAL conditions are initially different because of the different number of cycles until failure for each type of loading, the severity of degradation and the lifespan of the specimen can be predicted and evaluated by assuming that the total entropy generation is independent of the load and when the degradation coefficient of a material under CAL is given [31,71].

## 5. Conclusions

This study investigated the entropy generation in dual-phase steels during FCG and proposed the EGFC model. Additionally, an expression for entropy generation in terms of temperature evolution was developed using the concept of the DEG theorem. On the basis of the DEG theorem, the EGFC model was developed to predict the FCG rate. This theorem showed that entropy generation and crack growth are closely related because of the degradation coefficient *B*, allowing the easy determination of the empirical Paris–Erdogan law of crack growth from the DEG theorem’s considerations. Results showed that the EGFC model is in good agreement with the experimental results for dual-phase steels under spectrum loading conditions. The value of the RMSE in all cases is 10^−7^ m/cycle. Based on the results, the EGFC model is applicable in a variety of loadings, particularly those exhibiting a ∆*K*^2^ dependence on the FCG rate or for materials with *m* = 2. Furthermore, the predicted fatigue life under CAL and VAL conditions can predict the actual fatigue life obtained from the experimental work with an error less than 5%. Finally, the entropy generation calculated from the surface temperature of a specimen under FCG test can be utilised to predict the FCG rate of dual-phase steel via intensity degradation coefficient.

## Figures and Tables

**Table 1:** Chemical compositions of the steel (wt.%).

Elements	C	Mn	Si	P	S	Al
wt %	0.192	1.61	0.384	0.0162	0.0085	0.0314

**Table 2:** Mechanical properties of the steels.

Properties	Measured Value	
	As-Received	Dual-Phase
0.2% Yield strength (MPa)	388	495
Tensile strength (MPa)	536	597
Elongation at fracture (%)	30	13
Yield ratio (%)	72	83
Young’s modulus (GPa)	204	185

**Table 3:** Accuracy of the EGFC (entropy generation of fatigue crack) model with respect to the experimental data.

Loading	RMSE (m/Cycle)
CAL	1.0291 × 10^−7^
L-H	1.9769 × 10^−7^
H-L	1.5409 × 10^−7^

**Table 4:** CV values for FCG rate prediction.

Coefficient of Variance (CV)	FCG Rate (m/Cycle)	
	Proposed Model	Experiment
CAL	66.02%	61.87%
L-H	74.25%	51.65%
H-L	74.33%	65.61%

**Table 5:** Accuracy of the predicted fatigue life based on the experimental fatigue life.

Type of Load	Predicted Life _(EGFC)_	Experimental Life	Error (%)
CAL	53,535	53,674	0.3
L-H	115,592	112,683	2.6
H-L	128,407	123,056	4.3
